# The associations between religiosity and resilience when individuals are challenged by risk factors of suicide and mental illness

**DOI:** 10.3389/fpubh.2024.1442248

**Published:** 2024-07-31

**Authors:** Li-Jie Du

**Affiliations:** Sociology Department, Philosophy, Law, and Politics College, Shanghai Normal University, Shanghai, China

**Keywords:** religion, mental health, religiosity, spirituality, resilience, suicide, risk, mental illness

## 1 Introduction

As Bell says, the influence of religion should never be so insignificant that there is no functional alternative to religion (1). The inner experience of spiritual and religious emotions is indeed an important aspect of daily life for countless people around the world (2). For many people, spirituality and religion provide a framework for understanding the world, seeking guidance in times of difficulty, and connecting with something greater than themselves. These experiences can manifest in various ways, such as through prayer, meditation, rituals, and volunteers service actions. A general pattern emerging from a study analyzing psychosocial correlates across countries suggests that positive religious coping is most strongly associated with indices of positive functioning (i.e., post-traumatic feelings of growth and wellbeing), whereas negative religious coping is most strongly associated with indices of psychological distress (i.e., anxiety and depression) (3). Results also show that psychiatrists are more likely than other physicians to note that religion/spirituality sometimes causes negative emotions that lead to increased patient suffering (82 vs. 44%), and religion or spirituality can be damaging to mental health by means of negative religious coping, misunderstanding and miscommunication, and negative beliefs (2). The article which based on the published literature on the subject, emphasizes that associations between religiosity and the resilience when individuals are challenged by the risk factors of suicide and mental illness, mainly focused on the positive effect of religion on mental health and reducing suicidal ideation. Resilience can indeed be conceptualized as the ability to achieve positive, adaptive outcomes in the face of adversity (4). It involves the capacity to bounce back from difficult experiences, navigate through challenges, and emerge stronger and more resourceful.

Religiosity and spirituality are two concepts which are overlapping constructs, sharing some characteristics but also retaining non-shared features (5). In a study exploring the students' constructs of religiosity and spirituality, religiosity was portrayed as practice-oriented activity, with a faith in a divine or supreme being, often conducted within a religious community, that involved regulation of actions and behavior, while spirituality was associated with a faith in the existence of a higher power, a connection with something larger than the individual, and a deeper relationship with God or this higher power (6). Religiosity and spirituality are identified as different terms that shared common themes in this article. Also religiosity was chosen as the title because of more discussions on religious belief as the practice carried out within a religious community.

For over 100 years scholars from diverse fields such as psychology, sociology, public health, and religious studies have rigorously explored the intricate relationship between religious beliefs and the incidence of suicidal behavior (2). Mental illness has been identified in numerous studies as a significant contributing factor to suicide ideation (SI), and specific psychiatric symptoms, such as mood disorders, have also been recognized as predictors of suicidal thoughts (7). Nevertheless, the suicide and mental illness are regarded as two separate and crucial risk factors encountered by individuals in the modern society and will be examined independently within this paper. Accordingly, the suicide risk groups in the first section of this article exclude people with mental illness from its discussion.

## 2 Subsections relevant to the subject

### 2.1 The positive role of religious beliefs in preventing suicide in individuals

In sociological analyses, social integration, including religion and residential stability, is often considered a protective factor against suicide (7), where the concept of suicide is relevant to sociological perspectives, especially in the context of religious integration. Emile Durkheim's theory on the relationship between religion and suicide emphasizes the significance of religious integration, which revolves around the notions of shared beliefs and communal religious practices. According to this perspective, suicide is not considered a conscious choice made by individuals, but rather a consequence of external influences or forces. This sociological view provides valuable insights into the complex interplay between religious factors and the phenomenon of suicide, highlighting the role of shared beliefs and practices within religious communities. As Durkheim argued that the Catholic community has a lower suicide rate than the Protestant community due to its higher level of social integration is presented, and the greater sense of integration in Catholic communities is a stronger protective factor for individuals, which leads to a lower suicide rate than in Protestantism (8).

The existing research on the correlation between religion and suicide has primarily focused on the concept of religious integration as a causal factor (9). However, some researchers introduce an alternative perspective which is the concept of religious commitment, which posits that a strong commitment to life-affirming religious beliefs, values, and practices can effectively reduce suicide rates (10). Religious commitment, as assessed through fundamental beliefs and practices, emerges as a robust deterrent against suicide. For example, a conviction in an afterlife can serve as a motivational factor in enduring worldly hardships. The assurance of eternal salvation and heavenly glory provides solace to those enduring suffering, framing the adversity as transient compared to eternity and, consequently, potentially enhancing individuals' resilience in confronting challenges (11). Religion can significantly impact reducing suicide rates through rallying congregations against a common adversary such as Satan in Christianity, advocating prayer as a solution to adversity, and idealizing poverty, which may be associated with an increased risk of suicide (12). Buddhism highly values the importance of life and cherishes it, which does not advocate meaningless sacrifices, and believes that those who commit suicide or die unnatural deaths are not easily reincarnated and given a second life. It works to reduce inclinations toward social comparisons, thereby acting as a safeguard for happiness (13). But a few studies shows in Chinese MMT clinics, patients for heroin independence with Buddhist belief may have a high current suicide risk (14). Due to cultural and sample differences, it might be difficult to generalize Christianity findings to people of non-Christian countries and we need more evidence from the Buddhism research.

Individuals living in countries with high levels of religiosity, particularly those affiliated with one of four major faiths in the world (e.g., Christianity, Islam, Judaism, Buddhism) and displaying religious commitment and engagement with a religious network, tend to exhibit lower levels of acceptance of suicide (15). For instance, affiliation with Islam is specifically linked to reduced acceptability of suicide. These findings provide empirical support for the perspective of religious integration (16).

The beneficial effects of religiosity in reducing suicide risk are evident even in secularized European countries, where the reinforcing effects of religious moral values may be relatively attenuated (17). The association between religiosity and declining suicide rates observed in research findings confirms this hypothesis, suggesting that enhanced subjective religiosity is consistent with a reduction in the incidence of suicide. Further empirical investigations are necessary to elucidate this correlation in a variety of cultural contexts across the world (18).

### 2.2 Religiosity helps to develop the resilience in the rehabilitation of the people with mental illnesses

A large body of evidence suggests that spiritual and religious backgrounds, beliefs, and practices (SRBBPs) are associated with better psychological health. Professionals in the mental health disciplines, which include psychiatry, psychology, social work, marriage and family therapy, licensed professional counselors, certified chemical dependency counselors, and psychiatric mental health nurses, among others, encounter SRBBPs in their clinical practice (19). Research has demonstrated the influential role of religious and spiritual (R/S) beliefs on mental health, which shows that the religious and spiritual (+R/S) group exhibited notably higher levels of religious coping and lower levels of attachment insecurity, depression severity, and social anxiety in comparison to the non-religious/spiritual (–R/S) group (20). Some studies have demonstrated the variability in the impact of different religions on mental health: Christians who are highly involved in the organizational aspects of their religion report fewer depressive symptoms than Jews who have high levels of organizational religiosity, and the opposite is the case at lower levels of organizational religiosity (21).

The surveys indicated that North Americans frequently adhere to religious beliefs, and psychiatrists are more apt to encounter issues pertaining to religion and spirituality in clinical settings compared to other physicians (22). The conclusions drawn from these studies which investigated the correlation between religious or spiritual beliefs and severe depressive disorder, which utilized nationally representative longitudinal samples in Canada, suggest that regular attendance at religious services (at least once a month) has a protective effect against severe depression (23). The survey conducted by the California Mental Health & Spirituality Initiative in 2009 revealed that over 80% of the 2,050 participants agreed or strongly agreed on the importance of spirituality in relation to their mental health. Furthermore, respondents identified various spiritual practices that they found beneficial for their mental wellbeing, including prayer (73%), meditation (47%), attending religious services (40%), spending time in nature (41%), and reading sacred texts, or spiritual self-help books (36%) (24).

Clinical experience indicates that incorporating considerations of religion and spirituality can be crucial in the treatment process. There are growing efforts to examine religious and spiritual factors through the lens of developmental psychopathology, considering them as potential risk and protective factors for mental health disorders in children and adolescents (25). The failure to assess the client's spirituality has been identified as a barrier that hinders nurses from delivering adequate spiritual care, which is emphasized that incorporating spiritual therapy into nursing care can be effective if the client understands its importance, and if nurses who have a strong sense of spirituality are able to apply it effectively (26). This highlights the importance of recognizing and addressing spiritual needs in mental health care to ensure comprehensive and holistic support for individuals experiencing mental health challenges. Psychiatric mental health nurses could play a key role in helping patients with severe mental disorders in assisting individuals with severe mental disorders by addressing their spiritual needs, that acknowledgment of the importance of spirituality in mental health underscores the evolving understanding of comprehensive approaches to mental health care (27).

In a qualitative study examining the effects of religious practices on the mental health of Muslim patients diagnosed with schizophrenia, findings suggested that consistent adherence to rituals and recitations had a positive impact on the physical and mental wellbeing of inpatient individuals with schizophrenia, and psychiatric hospitals ought to consider providing adequate resources (robes and Qur'ans), to facilitate religious practices for these patients (28). This underscores the potential significance of integrating religious beliefs into the therapeutic environment for people with schizophrenia.

## 3 Discussion

### 3.1 The integrative role of religion in developing the individual's resilience against various risks should be given high priority

In the preceding literature review and discussion concerning spiritual responses to risk, the analysis emphasizes that resilience is best conceptualized as a process involving the interplay of multiple biological, psychological, social, and ecological systems.

Some studies explored the mechanisms through which the religious characteristics of communities are likely to influence individual behavior, which concludes that religion, despite being overlooked, represents a potentially significant cultural aspect of social organization within communities (29). The research seeks to investigate whether the religious attributes of groups and social contexts could effectively complement social disorganization theory by serving as a moral and social organizing force within a community (30). Additionally, it emphasizes the importance of fostering the social environment that promotes an inclusive understanding of diverse perspectives and values to contribute to comprehensive suicide prevention efforts. Drawing from previous research, it asserts that addressing the challenges of modern society necessitates the cultivation of improved interpersonal relationships, trust, mutual assistance, as well as a sense of belonging and solidarity in the community. The majority of the globe's population engages in RS practices, which are intertwined with their cultural identity (31). While studies investigating the relationship between religion, spirituality and mental health often produced positive associations (32), we recognize the need for a more refined approach, with careful differentiation between cultures and traditions, and a greater focus on the contextual experiences of individuals within particular traditions (33).

Furthermore, resilience is not a fixed trait but rather a dynamic process that can be nurtured and developed over time (34). This can include developing problem-solving skills, seeking support from others, maintaining a positive outlook, and adapting to changing circumstances (35). Religion serves as an intrinsic support for individuals, aiding in their understanding of life and death. Each religious system offers unique perspective and meanings, while most of them considering death as a natural phase in human development instead of being forced to die adversely in the face of adversity. By understanding resilience in this way, researchers can design and implement interventions aimed at developing specific skills and attributes that contribute to positive adaptation to the life adversity (36).

### 3.2 The ethical and moral issues surrounding the integration of religious or spiritual perspectives into the practice of psychiatry are still being debated

Contemporary psychiatrists increasingly recognize the significance that integrating religion and spirituality into treatment. However, persistent complex ethical issues arise, particularly regarding the ethical justification for psychiatrists to engage in religious or spiritual practices with their patients or operate from a specific religious or spiritual perspective. This is especially pertinent in situations where it is illegal to openly advertise their adherence to specific religious or spiritual beliefs.

These issues demand careful consideration within the context of mental health care. Psychiatrists must navigate these questions while upholding professional boundaries and demonstrating sensitivity to the diverse beliefs and backgrounds of their patients. It is essential to maintain ethical standards and ensure an inclusive, non-discriminatory approach to care. The researchers present the implications of a bio-psycho-social-religious/spiritual model for psychological development and functioning (37). This shift highlights the need for a more comprehensive understanding of how religious and spiritual factors intersect with mental health, offering potential insights into holistic approaches to psychiatric care that consider the influence of belief systems and existential inquiries on individual experiences of mental wellbeing.

Throughout the history of psychiatry in the United States, the importance of religious and spiritual dimensions in mental health care has been consistently acknowledged. In the 19th century, religion and moral therapy were intricately linked, becoming foundational elements of psychiatric hospital treatment frameworks. In the 20th century, psychoanalysis rose to prominence within the psychiatric field, prompting some psychiatrists to engage with the nascent discipline of pastoral care. This historical evolution underscores the ongoing rediscovery and acknowledgment of the interplay between religion, spirituality, and mental health (38). The resolution of these nuanced ethical issues requires ongoing dialogue and reflection within the mental health community to ensure that religion and spirituality are integrated into treatment in a responsible and respectful manner. These insights offer valuable implications for further research into treatment strategies that evaluate the role of spirituality/religion and integrate protective elements of spirituality/religion into mental health interventions (39).

## Author contributions

L-JD: Conceptualization, Data curation, Formal analysis, Funding acquisition, Investigation, Methodology, Project administration, Resources, Software, Supervision, Validation, Visualization, Writing – original draft, Writing – review & editing.
